# Characterization of a mine legacy site: an approach for environmental management and metals recovery

**DOI:** 10.1007/s11356-019-06987-x

**Published:** 2020-01-23

**Authors:** Maria de Lurdes Dinis, António Fiúza, Aurora Futuro, Alexandre Leite, Diogo Martins, Janine Figueiredo, Joaquim Góis, Maria Cristina Vila

**Affiliations:** 1 Center for Natural Resources and the Environment (CERENA-FEUP), Faculty of Engineering, University of Porto, R. Dr. Roberto Frias, 4200-465 Porto, Portugal; 2 Faculty of Engineering, University of Porto, R. Dr. Roberto Frias, 4200-465 Porto, Portugal

**Keywords:** Mine tailings, Arsenic, Acid mine drainage, Waste management, Reprocessing

## Abstract

The characterization of historical mine tailings provides important information for land-management decisions, in particular when considering potential reprocessing activities or the development of an environmental protection program. In addition, outcomes from such characterization may define the scope for a more detailed investigation. The present work describes the characterization of the waste material from the Cabeço do Pião tailings impoundment performed within the project ReMinE: Improve Resource Efficiency and Minimize Environmental Footprint. The purpose of the work was to investigate alternative mine waste management options such as the extraction of valuable resources from an environmental liability. The study involved the collection of 41 samples at different locations at two different depths, physical and chemical characterization of the wastes, natural leaching tests, and potential for acid generation. The results showed that, apart from the potential instability of the dyke (with an average slope of 35°), the drained solutions flowing by percolation contain very small particles with high arsenic contents that are being incorporated into the river sediments. In addition, these very fine-grained materials are available for the transport by the wind creating secondary sources of environmental contamination. This data is fundamental for economic and environmental assessment of the two main alternatives, reprocessing or removal.

## Introduction

Mine waste (waste rock and tailings) management still represents a challenge for many countries where mining activities were, or still are, in place. In particular, historical mine wastes can constitute a threat or an opportunity for local communities. They become a threat when they are simply abandoned and measures are not taken to reduce the risks to the environment but they can also represent an interesting resource of critical and valuable metals with potential economic benefits where the reprocessing may reduce environmental liabilities both for the public and private stakeholders. In many cases, the deposits of historical mine wastes, derived from past mining and metallurgical activities, have recoverable grades for present standards and, in certain cases, significant amounts of other potentially valuable metals (Bellenfant et al. 2013).

Therefore, any new extractive waste treatment process should be accompanied by information about the physical and chemical characteristics of the waste in order to provide the required useful information to the authorities and to the companies that are intending to begin with potential reprocessing activities or with an environmental protection program. The mitigation of the environmental impacts resulting from mine wastes, and the remediation options, will depend on the waste characteristics and its behavior in the environment.

Mine tailings may be highly reactive due to their small particle size and content of reactive minerals, such as pyrite (FeS_2_). Other metals that may be present include base transition metals such as iron, copper, nickel, and zinc, in relatively high concentrations, and occasionally precious metals such as gold and silver. Toxic elements, such as arsenic, may also be present in high concentrations.

The exposure of reactive mine tailings to both oxygen and water can generate acidic effluents and runoff waters containing high concentrations of dissolved metals and sulfates. Acid mine drainage (AMD) is a widely reported common environmental impact at mine sites. For historical mining waste deposits, the geotechnical stability and the potential release of dissolved metals, acidity, or suspended particles can also be a serious and long-lasting problem (INAP 2019, Tremblay and Hogan 2001, Lottermoser 2010).

The Cabeço do Pião tailings impoundment was considered as a case study of historical mining wastes for the present work. The interest in reprocessing the tailings coexists with the necessity to solve the environmental problems caused by the potential instability of the unconfined tailings if left exposed during severe meteorological conditions. This impoundment is located on a bank of the Zêzere River and its materials have an average arsenic content of 15%. Other metals (e.g., Cu, Zn, and W) are present with significant concentrations.

The present work describes the characterization of tailings from Cabeço do Pião performed within the ERA-Min project: Improve Resource Efficiency and Minimize Environmental Footprint (ReMinE). The study involved the collection of 41 samples at different locations at two different depths, physical and chemical characterization of the wastes, natural leaching tests, and screening tests for acid generation potential.

## Materials and methods

### Study area

**Fig. 1:**
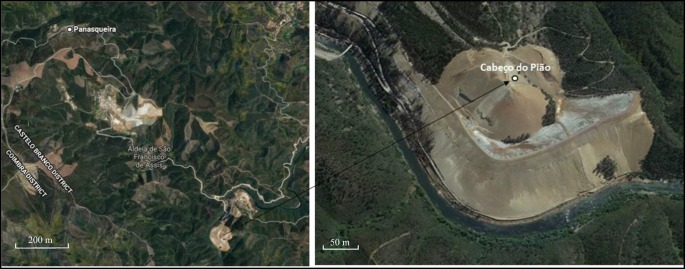
Panasqueira mine and Cabeço do Pião tailings deposit

The Cabeço do Pião tailings impoundment belonged to the Industrial Complex of Panasqueira mine (Fig. 1), which is one of the largest operating tungsten mines in the Market Economy Countries (MEC). The Panasqueira mine started operating in 1896 focusing mainly on wolframite exploitation with cassiterite and chalcopyrite exploitation as by-products (Candeias et al. 2014).

The geology of the Panasqueira mine was extensively studied and described by several authors (D’Orey 1967; Bloot and Wolf 1953; Kelly and Rye 1979; Noronha et al. 1992; Ávila et al. 2008). The ore deposit is considered one of the biggest W-Sn deposits of Western Europe and it is located in the Central Iberian Zone (CIZ). The deposit is a classic example of a W-Sn hydrothermal mineralization associated with the Hercynian plutonism. The present minerals include quartz, wolframite, pyrite, pyrrhotite, arsenopyrite, chalcopyrite, cassiterite, beryl, mica, and fluorite (Candeias et al. 2014; D’Orey 1967; Bloot and Wolf 1953; Kelly and Rye 1979; Noronha et al. 1992; Ávila et al. 2008; Grangeia et al. 2011). Additionally, many rare minerals were identified, including sulfides, sulfosalts, oxides, carbonates, silicates, phosphates, and tungsten (Kelly and Rye 1979).

**Fig. 2:**
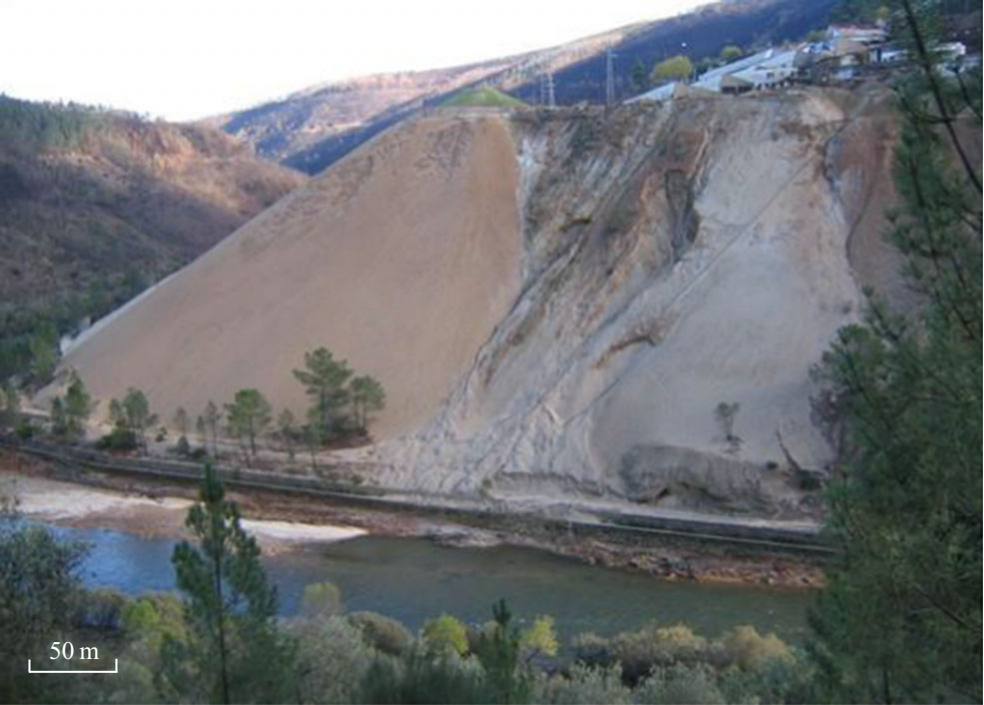
Slope of the Cabeço do Pião tailings deposit

At the beginning, the mining scale was very small, but it increased until 1928 and finally became a large exploitation. The Cabeço do Pião was one of the seven areas where the exploitation took place. The wastes were produced by one of the three processing plants that existed in the mine, designated as Rio or Cabeço do Pião Processing Plant. The resulting tailings were therefore disposed of at the Cabeço do Pião site, in an impoundment around a hill on the edge of the Zêzere River, starting in 1927 and continuing for 90 years. The slope of the crest and supporting bedrock is in average 35° and the average height is approximately 90 m, draining directly to the Zêzere River (Fig. 2).

The total estimated volume of the tailings dam is 730,000 m^3^. The tailings are exposed to atmospheric conditions and altered by chemical, mineralogical, physical, and geotechnical factors. In addition, an arsenopyrite stockpile (9400 m^3^) was deposited near the former processing plant and remained exposed until 2006, when it was capped with geotextile and layers of clay (Candeias et al. 2014).

The altitude in the region ranges from 350 to 1080 m forming deep valleys. The Zêzere River is the main watercourse in the area. The climatic conditions can be extreme, with rainy and windy winters and very dry and hot summers. The average annual precipitation is 1600 mm and snow events occur frequently above the altitude of 700 m. The average temperature ranges between 0 °C in the winter and about 30 °C in the summer (Candeias et al. 2015).

Collecting representative samples of mine waste material can be challenging due to the high heterogeneity of the chemical and mineralogical composition of the tailings. Different origins of the ores that were processed, as well as different production techniques over time, and different deposition strategies influenced the characteristics of the tailings such as the particle size distribution, type of the secondary minerals, and concentration in heavy metals (Martin et al. 2016).

The tailings from Cabeço do Pião were sampled at 41 sampling points for surface samples (50 to 60 cm depth) and for deep samples (approximately 2 m) with a mechanical shovel. Surface samples are most relevant for wind-borne transport, exposure, and direct contact with precipitation and surface runoff.

**Fig. 3:**
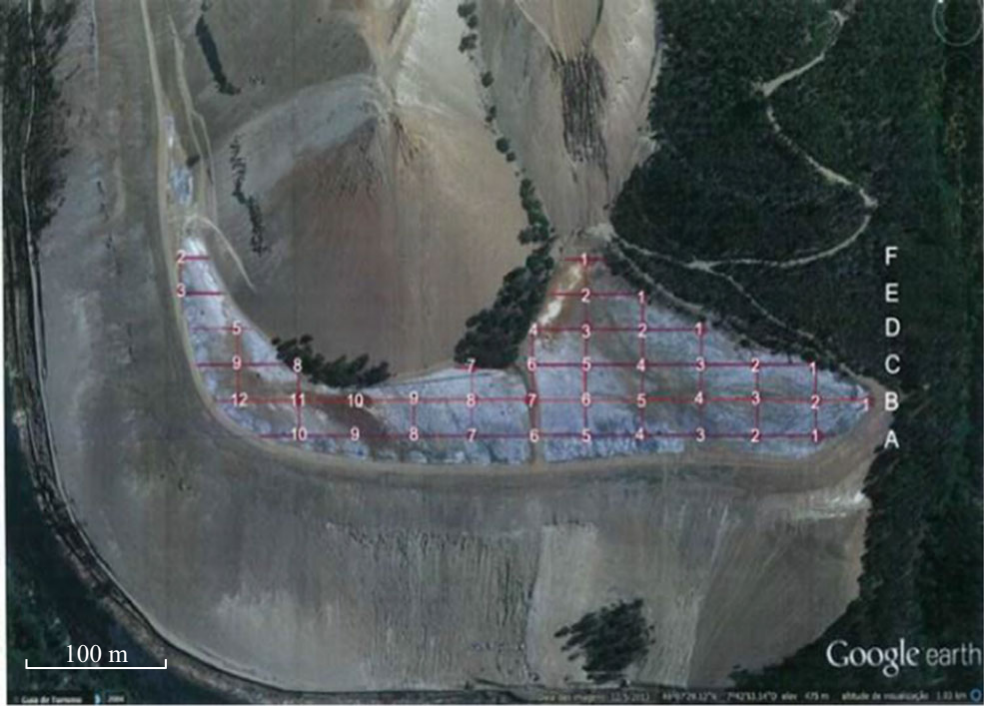
Cabeço do Pião sampling grid (adapted from Google earth, 2016)

The sampling events took place between November 2016 and January 2017. A rectangular grid of 40 × 20 m (Fig. 3) was defined for the collection of samples. The samples were identified with letters from A to F followed by a numbering sequence from the right to the left side of the figure. In addition, the samples were geo-referenced and identified as “S” for the surface samples and as “P” for the deep samples. A few samples were selected for a more detailed characterization within different studies and purposes (Figueiredo et al. 2018a; Figueiredo et al. 2018b; Albuquerque et al. 2017).

### Experimental methods

The experimental methods included determination of particle size distribution, chemical composition, pH, density, and porosity.

Tailing samples were oven-dried at 60 °C until constant weight (the weight was constant for two consecutive readings after 48 h), homogenized, and sieved through a 200-mesh screen. The grain size distribution of the undersize fraction was analyzed with a laser diffraction particle size analyzer (Malvern Mastersizer 2000).

The bulk chemical composition was determined by X-ray fluorescence (XRF) with an Oxford XRF Analyzer (X-MET 7500). The consistency of the analysis was verified by using samples with known grades (measured in a wavelength dispersive X-rays analyzer) that were re-analyzed in the X-MET 7500 analyzer. A very good fitting between both assessments were achieved and not only for tungsten but also for all other elements analyzed. Therefore, this equipment was used for the analysis of the chemical composition of the solid samples, using the specific calibration curve obtained specifically for the assessment of tungsten.

The pH of the tailings was determined in an aqueous solution by two different methods, with distilled water in a 1:1 solid-liquid ratio and with 0.01 M CaCl_2_ in a 1:2 solid-liquid solution ratio (Black 1965). Particle densities were determined by the pycnometer method described in the standard CEN ISO/TS 17892-3:2004.

Natural leaching tests were performed with representative tailing samples. The leaching process was simulated with distilled water in batch cells with constant stirring for a maximum period of 24 h. The tests were stopped at 1, 3, 12, and 24 h to measure the pH, dissolved oxygen, salinity, total dissolved solids, conductivity, and temperature. The standard method DIN 38414-S4 was followed to set up the experimental phase in the laboratory. The leached solid samples were dried and then analyzed for chemical composition by XRF. The results were compared with the chemical composition of the unleached samples, given in mass percentage.

The acid generation capacity was also tested using two acid generation prediction methods: the Net Acid Generation test (NAG) and the modified Acid Base Accounting (ABA) method for neutralization potential (Lawrence and Wang 1997). Ancient mine wastes are weathered material and therefore, a method that incorporates the potential acid production of secondary and tertiary minerals and the potential acid-consuming capacity of host rock minerals (e.g., carbonates, aluminosilicates, and silicates) should be adopted (Smith et al. 2006). Six sampling sites were randomly chosen from the 80 different locations defined in the sampling grid. The material of these samples was in turn homogenized and resampled using a Jones Riffle Splitter.

The NAG test determines the balance between the acid producing and acid consuming components of tailings/waste rock samples. NAG results provide the acid rock drainage characteristics based on the complete oxidation of the sulfide content of the samples (as well as ferrous iron from siderite dissolution). Acid that is produced by oxidation is consumed by carbonates and/or other acid-consuming components of the material. The pH of the solution is measured (NAG pH). The acid remaining after the reaction is titrated with standardized NaOH to determine the net acid generated.

The modified acid–base accounting (ABA) (Lawrence 1990) determines the maximum potential for acid production (acid production potential or APP) and acid neutralization (neutralization potential or NP). The procedure involves a laboratory static test that compares the maximum APP of a sample with its maximum NP. The APP is based on sulfur analysis, and the NP is determined by the amount of acid neutralized when the sample is in contact with a solution in the approximate pH range of 1 to 3.5. The potential of a mine waste to produce acidic drainage is determined by the difference (net NP) or ratio (NP/APP) of these values (Lapakko 1992).

## Results and discussion

### Characterization of tailing samples

#### Physical properties

**Fig. 4:**
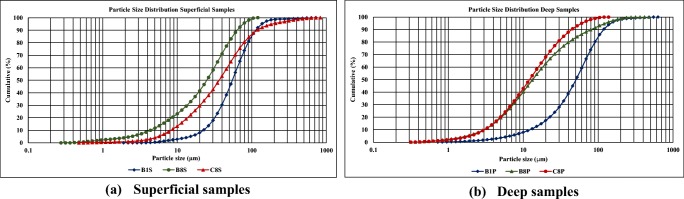
Particle size distribution of tailing samples from Cabeço do Pião impoundment. **a** Superficial samples. **b** Deep samples

The grain size distributions of the mine tailings collected are presented in Fig. 4. Two different depths were considered: 0–0.5 m and 0–2 m, and are designated as surface (S) and deep samples (P), respectively.

The mine tailings are composed of particles ranging in size from 0.25 to 900 μm for surface samples and particles ranging in size from 0.3 to 700 μm for deep samples. The overall composition includes sand-sized particles ranging in size from 62 to 1000 μm, silt-sized particles ranging in size from 4 to 62 μm, and clay-sized particles < 4 μm. The following observations emerge from the grain size analyses for the surface and deep tailing samples.

The surface samples are very heterogeneous with regard to grain size. The silt fraction represents the highest content: 65%, 84%, and 73% for B1S, B8S, and C8S, respectively. The sand content is 34%, 8%, and 24% for samples B1S, B8S, and C8S, respectively. The *D*-values (D10, D50, and D90) are as follows: B1S (4 μm, 25 μm, 70 μm), B8S (8 μm, 35 μm, 100 μm), and C8S (2 μm, 65 μm, 100 μm).

For the deep samples, the silt fraction presents the higher content: 57%, 73%, and 82% for B1P, B8P, and C8P, respectively. The sand content is 41%, 16%, and 7% for samples B1P, B8P, and C8P, respectively. The *D*-values (D10, D50, and D90) are as follows: B1P (13 μm, 52 μm, and 102 μm), B8P (3 μm, 13 μm, and 80 μm), and C8P (3 μm, 12 μm, and 46 μm), respectively.

During industrial operation, there were two sources of fine tailings: the overflow of a classification by hydro-cyclones and the depressed tailings of flotation. Both were discarded using moving inlet points. The grain size stratification of the disposal in depth is probably due to preferred sedimentation of coarser and finer particles. The existence of grain size variations within the impoundment where the former general rule is disrupted derives from the existence of multiple inlet points: the coarser grains settled closer to the pipe, while the finer grains settled further away.

For surface samples, the loose bulk density ranged from 1.10 to 1.54 g/cm^3^ and the compacted bulk density ranged between 1.83 and 2.50 g/cm^3^; the particle density ranged from 3.16 to 3.76 g/cm^3^, and the voids percentage ranged from 35 to 41%. For the deep samples, the loose bulk density ranged between 1.07 and 1.46 g/cm^3^ and the compacted bulk density ranged between 2.14 and 2.44 g/cm^3^; the particle density ranged between 3.58 and 3.89 g/cm^3^ and the voids percentage ranged from 37% to 41%. The tailings exhibit low bulk densities and there is no significant variation with depth; the same pattern was verified for the variation of particle density with depth. Nevertheless, the void percentage slightly decreased for deeper samples evidencing some consolidation, simultaneously with the transport of very fine particles partially occupying the void pores.

#### Environmental properties

**Fig. 5:**
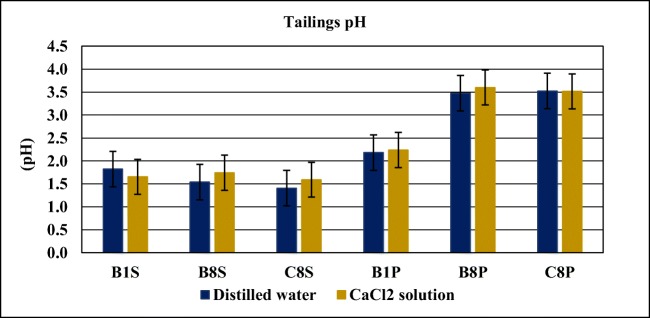
Tailings pH determined in solution with distilled water and CaCl_2_ solution

The pH of the tailings measured in solution with distilled water and CaCl_2_ is presented in Fig. 5. The difference of the results between the two methods can be neglected.

All samples show acidic pH values ranging from 1.4 to 3.6. However, the surface samples (S) present a lower pH than deep samples (P). The higher acidic pH observed at the surface is apparently a consequence of atmospheric oxidation of the exposed pyrite minerals in the tailings deposit that produces sulfuric acid (acid mine drainage).

#### Chemical composition

A summary of the chemical composition of the tailing samples is presented in Table 1.

**Table 1:** Chemical composition of the tailing samples from Cabeço do Pião impoundment (mass %)

Element	B1S	B8S	C8S	B1P	B8P	C8P
As (%)	13.10	17.87	12.81	18.75	14.17	12.39
Cu (%)	0.36	0.09	0.70	0.79	0.51	0.56
Fe (%)	24.11	20.98	21.22	27.25	27.03	26.13
Hg (%)	0.03	0.06	0.04	0.04	0.04	0.05
K (%)	0.55	0.34	0.64	–	–	–
Mn (%)	–	–	–	0.01	0.21	0.21
Sb (%)	–	–	–	–	0.01	0.01
Sn (%)	0.05	0.10	0.07	0.08	0.09	0.08
S (%)	9.90	2.50	10.10	10.40	7.40	7.50
W (%)	0.06	0.25	0.23	0.15	0.47	0.50
Zn (%)	0.82	0.05	0.64	1.26	1.63	1.56

**Fig. 6:**
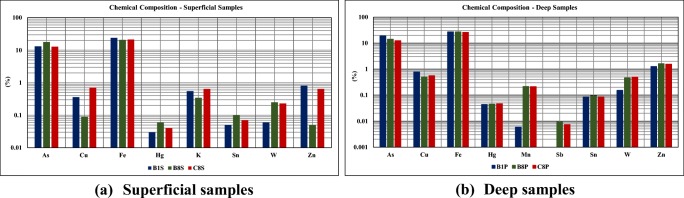
Chemical composition of tailing samples from Cabeço do Pião impoundment. **a** Superficial samples. **b** Deep samples

The results show the prevalence of Fe (≅ 25%) and As (12–20%), followed by Zn (0.99%), Cu (0.5%), and W (0.28%). There is an observed increase in Fe, Mn, Zn, and W content with depth and a decrease in K content with depth for all samples. Although K is soluble and can easily percolate down in the tailings profile, however, the simpler compounds decompose in hot water while many of the common K minerals are listed as insoluble in the range of 0–100 °C. The coexistence of potassium compounds such as feldspar, mica, and silicates is responsible for the peculiar behavior in the tailings surface and depth environments where the potassium is bond to an insoluble or relatively insoluble mineral form (Grangeia et al. 2011). The As content increases with depth for sample B1 but decreases for samples B8 and C8, while Sn content increases with depth for samples B1 and C8 and decreases for sample B8. The results show that the major environmental concern is the high As content present in the tailings. The oxidation of sulfide minerals exposed to atmospheric oxygen can generate acidic drainage when in contact with water. The overall results are presented in Fig. 6.

In general, the average results of all tailing samples showed some important and interesting elements: As = 143,041 mg/kg; Cu = 4738 mg/kg; Fe = 23.84%; W = 2496 mg/kg; and Zn = 99,944 mg/kg. The relative standard deviation is between 0 and 5% (Figueiredo et al. 2018a).

In previous studies, the materials from the Cabeço do Pião impoundment were characterized from selected samples from drill cores (Grangeia et al. 2011). For all core samples, As is enriched from the surface down to a depth of 13 m, with a concentration ranging between 8.7% and 24%. From this depth, arsenic content decreases to values near or below 1%. Results from X-ray diffraction (XRD) analyses described in Grangeia et al. (2011) showed the presence of quartz, mica, feldspar, ilite-vermiculite, arsenopyrite, marcasite, pyrite, pyrrhotite, and chalcopyrite. Other minerals, like scorodite and natrojarosite, are also present and enriched in As, Cu, Mn, Pb, and Zn.

The fine-grained nature of these materials with high As content is the most immediate environmental concern both due to the potential transport and dispersion by the wind and the proximity to the Zêzere River. Moreover, other elements are present at high concentration in particles that are readily mobilized during ongoing removal and processing. The material from the arsenopyrite stockpile that was deposited on the top of the tailings contains high concentrations of Ag (124 mg/kg), As (210,000 mg/kg), Cd (3057 mg/kg), Cu (1426 mg/kg), Fe (19.8%), W (5166 mg/kg), and Zn (460 mg/kg) (Candeias et al. 2014; Ávila et al. 2008).

### Natural leaching tests

Natural leaching tests are one of the commonly used procedures worldwide to evaluate, characterize, and prioritize mine wastes. These tests allow identifying and estimating the amount of soluble constituents that can be released from waste rock materials and tailings during natural climatic conditions, which may include severe rainstorms and snowmelt events. Using this test, it is possible to determine the concentration of contaminant(s) that are present in the solidified/stabilized (S/S) waste form, and more significantly, their likely mobility.

For the collected samples, natural leaching tests were carried out as short-term extraction tests (24-h batch extraction tests using distilled water). These tests provide information on the short-term metal leaching potential, although in natural conditions (under the influence of atmospheric conditions and the presence of microorganisms), there are several physical, chemical, and biological processes that are not possible to reproduce in batch laboratory tests. The variation of leachate parameters overtime was also assessed.

#### Variation of pH over time

**Fig. 7:**
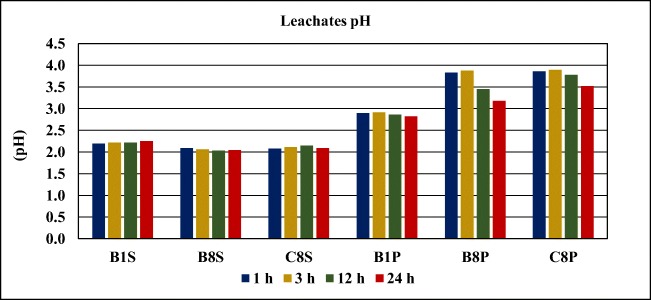
Variation of leachates’ pH over time

The pH of the leachate solution as a function of weathering (leaching) time in hours is presented in Fig. 7. At the beginning, the pH of the distilled water used to leach the samples was 6.6 for surface samples and 7.2 for deep samples. Typically, distilled water has a weakly acid medium and its pH is typically between 6 and 7, due to the dissolved carbon dioxide. The exact pH value basically depends on how it is stored and what the ambient conditions are as the moment it comes in contact with air, CO_2_ gas begins dissolving into it, forming carbonic acid.

The test results show a significant decrease in the pH of the leaching solutions (6.6 and 7.2) to values similar to those of the collected samples’ pH range (1.98–2.32). These are typical pH values of impacted waters resulting from the percolation of rainwater through the tailings, causing the weathering of the sulfide-rich minerals. During the sampling events, it was possible to observe the occurrence of acid drainage at the base of the tailings embankment, and the consequent development of iron coating and ferruginous crust. The chemical composition of these waters presents high concentrations of dissolved sulfates, Al, As, Cd, Co, Cu, Fe, Mn, Ni, and Zn which are indicative of the oxidation and dissolution of sulfides (pyrite, chalcopyrite, sphalerite, and arsenopyrite) (Grangeia et al. 2011). In addition, for pH values below 5, it is assumed that a potential aquatic toxicity exists from cationic metals (Al, Cd, Cu, Ni, Pb, and Zn) as in this condition, metals are generally dissolved and minimally complexed with organic or inorganic ligands (Smith et al. 2006).

#### Variation of salinity over time

**Fig. 8:**
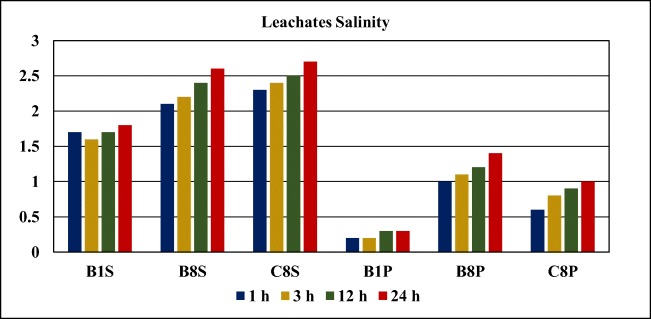
Variation of leachates’ salinity over time

The measured values for salinity during the leaching test are presented in Fig. 8.

The dynamics of salinity in tailings is very important for future revegetation of the disposed tailings as high salinity is one of the major constraints to revegetation in many tailings impoundments (Gozzard et al. 2009; Huang et al. 2012). Pyrite oxidation is a major factor responsible for the acidification of mine wastes, causing extreme salinity and metal toxicity (Evangelou and Zhang 1995). In general, areas requiring reclamation will be affected by high salinity of the generated leachates and therefore, when considering this option, an assessment of the tolerance of plants to salinity in runoff/seepage water is made by calculating the average root zone salinity (DITR 2018).

For the samples from the Cabeço do Pião tailings impoundment, the salinity increases over time. This results from the weathering of reactive tailings’ minerals, which acidifies the pore water and increases the release of saline ions and toxic elements. The tailings leachate drains directly to the Zêzere River.

#### Variation electrical conductivity over time

**Fig. 9:**
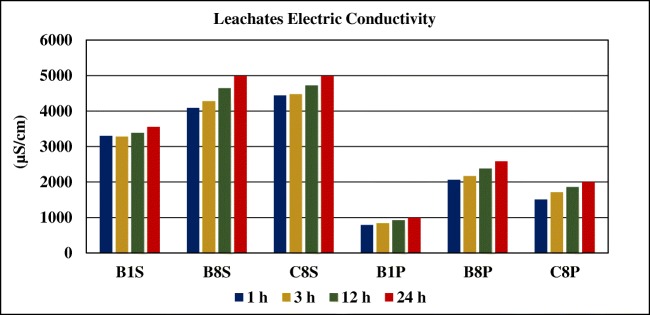
Variation of leachates’ electric conductivity over time

The results obtained for electrical conductivity (EC) during the leaching tests are presented in Fig. 9. As expected, both parameters, electrical conductivity and salinity, show a similar behavior over time. The values increase and are higher for surface samples (more oxidizing environment) than for deep samples. The increase in electrical conductivity and the decrease in pH indicate the tailings oxidation activity.

#### Chemical composition of the solid leached samples

**Fig. 10:**
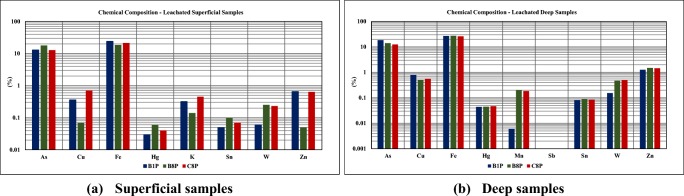
Chemical composition of the leached solid samples (mass %). **a** Superficial samples. **b** Deep samples

The leached solid samples were dried at 60 °C until constant weight (the weight was constant for two consecutive readings after 48 h) and analyzed on X-ray fluorescence for chemical composition. The results are presented in Fig. 10.

Apart from a few exceptions (Cu, Fe: B8S, C8S samples; K: B1S, B8S, C8S and Zn: B1S, B8P C8P), the chemical composition of the solid phase after the natural leaching test is similar to the chemical composition before the natural leaching test. Although the leachate presents an extremely acidic pH and the EC and salinity increase over time as well, which means high concentrations of dissolved sulfates, Al, As, Cd, Co, Cu, Fe, Mn, Ni, and Zn (Grangeia et al. 2011), it appears that there are some mechanisms that limit the effects of the acid drainage since the crusts and clays efficiently fixate some of the contaminant elements, especially the iron oxides (Ávila et al. 2008).

On the other hand, the mine tailings used in this study were sampled from historical mine tailings which means that the surface of the uncovered mine tailings had been allowed to be in contact with oxygen. This condition resulted in the oxidation of sulfide minerals. The oxidation of sulfide, which combines with As or heavy metals, results in the dissolution of As and heavy metals by water (Kim and Jung 2004). Based on the presence of sulfide minerals, high contents of As and heavy metals, potential of oxygen contact, and high sulfate concentration in the mine tailings, the dissolution of iron and arsenic, as an example, could have already occurred in the mine tailings. Heavy metals released by oxidation can be re-adsorbed onto the surface of ferric hydroxide and adsorbed heavy metals can be separated from the surface of ferric hydroxide by water (Holmstrom et al. 1999). The low leaching concentration in the test may also indicate that the more soluble fraction of As and heavy metals might have already been released by the oxidation of sulfide minerals with air and water for a long period at the tailings deposition site. Based on this, investigations on the status of contaminants in nearby soils and groundwater are needed.

### Acid generation potential

The static geochemical tests, NAG and modified ABA (Lawrence), were performed to determine the total acid-generating (AP) and total acid-neutralizing potential (NP) of the collected samples. Individually, each test has limitations on how accurately it can predict AP and NP and therefore, it is a good practice to use a combination of methods to define NPR (neutralization potential ratio: NP/AP) and identify samples requiring further investigation (Albuquerque et al. 2017).

#### Net acid generation test

The NAG test was performed on 2.5 g of pulverized sample (less than 75 μm); however, as the obtained NAG values were above 25 kg H_2_SO_4_/t (tons of acidity per ton of rock) for all samples, the test was repeated with 1 g of material as shown in Table 2.

**Table 2:** NAG test results

Samples	NAG pH	NaOH	NAG values
B1S	2.12	22.6	58.21
B1P	2.22	20.1	44.69
B8S	1.98	23.0	91.14
B8P	2.32	22.5	54.88
C8S	2.10	20.	61.94
C8P	2.24	25.0	52.14

In this test, hydrogen peroxide is used to accelerate the oxidation of sulfide (Lawrence et al. 1988). The reaction generates acid which in turn reacts with the buffering minerals in the sample. One potential limitation of this test is that if the extent of oxidation in the field setting is greater than in the test, the potential exists for the test to underestimate acid production, creating the possibility that some acid producing waste may be incorrectly classified as non-acid-producing (EPA 1994). This is not the case, as the results indicate that these samples have high risk of acid generating and therefore are classified as “potentially acid forming” (PAF) according to the Geochemical Classification Criteria based on NAG value and NAG pH (DITR 2018).

#### Modified Acid–Base Accounting Test

In this test, the acid production potential (APP) is determined from the sulfide sulfur content as follows: 31.25 × percent S = APP (modified ABA: the sulfur contribution from non-sulfide sources is not included) and assumes that two moles of acid will be produced for each mole of sulfur. Units for APP are tons of acidity per ton of rock. Neutralization Potential (NP) is determined first by a simple fizz test to select the acid strength to use in the next step to insure the addition of sufficient acid to react all the calcium carbonate present.

For the modified ABA test, 0.5 g of sample was first used to determine the fizz rating and then 2.0 g of sample (less than 250 μm) to determine the NP in order to calculate the NNP and NPR (Lawrence et al. 1988, Lawrence 1990, Miller et al. 1997). The fizz rating was classified as “none” for all samples. The results of the modified ABA test are presented in Table 3.

**Table 3:** Modified ABA test results

Sample	pH	NaOH (ml)	NP (kg CaCO_3_/t)	SO_3_ (%)	S (%)	AP (kg CaCO_3_/t)	NPP (kg CaCO_3_/t)
B1S	8.51	21.6	− 53.25	24.6	9.9	3.08	− 56.33
B1P	8.40	12.8	− 31.95	26.0	10.4	3.25	− 34.75
B8S	8.36	23.1	− 57.00	6.1	2.5	0.77	− 57.77
B8P	8.30	20.4	− 50.90	18.6	7.4	2.33	− 52.33
C8S	8.33	21.1	− 52.00	25.1	10.1	3.14	− 55.14
C8P	8.34	20.9	− 52.15	18.7	7.5	2.34	− 53.59

The modified method assumes that sulfur present as sulfate is not acid producing and therefore may underestimate available APP if jarosite or other acid-producing sulfate minerals are present. Conducting the acid digestion at standard temperature may reduce the contribution of iron carbonate minerals when determining the NP.

Assumptions of the test are that all the sulfur in the sample is reactive. This assumption does not take into account the presence of gypsum and other non-reactive sulfur minerals. A shortcoming of this technique is the potential to overestimate NP.

Tests conducted by Ferguson (Lapakko 1993) indicate that NNP values less than − 20 (kg CaCO_3_/t) are likely to form acid. Those with NNP values greater than 20 were not likely to form acid. For NNP values between − 20 and 20, it was difficult to determine the acid potential (samples are classified as “uncertain”). In this case, all samples are likely to form acid, as the NPP is less than − 20 (kg CaCO_3_/t). Also, a sample is classified as “possibly” or “likely acid generating” if the NPR of the samples is less than 1, which seems to be the case.

The modified ABA results, along with NAG results, indicated that these samples would produce acidity (PAF) and therefore, these historical mine wastes are a source of potential acidity that can be generated under natural oxidation processes.

The discharge of acid mine drainage with low pH and high dissolved metal content will seriously affect the aquatic environment (Grangeia et al. 2011).

### Reprocessing investigations and environmental alternatives

The reprocessing of zinc from the tailings of Cabeço do Pião deposit could represent a promising solution to the depletion of zinc ores worldwide. Zinc ores can be concentrated by acidic leaching processes, but this treatment presents the disadvantage of dissolving other metals, such as Fe, Ca, Mg, and Si. Several leaching tests were already performed with sulfuric acid and hydrochloric acid-oxygen leaching. Alkaline leaching tests of tungsten were also performed.

The reprocessing research was based on the following progressive steps: (a) zinc recovery by sulfuric acid atmospheric leaching; (b) froth flotation of arsenic in the leaching residue; and (c) pressure leaching of tungsten on the tails of flotation, having in mind that copper would not be recovered.

First, the reprocessing of zinc from the tailings of Cabeço do Pião was based in atmospheric leaching by acidic reagents. A plan of tests was developed to consider the effect of the following variables: type of reagent (H_2_SO_4_ or HCl), temperature (20, 50, or 80 °C), solid percentage (10, 20, and 40%), concentrations and composition of the leaching solution, and addition of oxidant. All the tests were performed at atmospheric pressure, using the same stirring speed (225 rpm) and the same residence time (6 h). Kinetics was studied collecting samples of the liquid phase after 1, 2, 4, and 6 h. The higher extraction yields (60%) were obtained for sulfuric acid, at a temperature of 80 °C, a pulp density of 40% of solids, using the following concentrations H_2_SO_4_ 0.5 M + Fe_2_(SO_4_)_3_ 0.5 M and with addition of oxygen (60 L/h).

The flotation of As was studied by performing a 23 factorial test, using three parameters (type of collector, its dosage, and froth bed height), considering two levels for each one. During these tests, the solid percentage was 30% Danafloat 507E (Danafloat) and Maxgold 900 (Cytec) used as collectors, being the first variable of the factorial test. Then, the collector dosage was varied—30 g/ton in the roughing stage and 15 g/ton in the scavenging stage vs 45 g/ton in the roughing stage and 22.5 g/ton in the scavenging stage. Finally, the froth bed height was varied between 3 and 6 cm. The factorial tests were carried out on a Leeds flotation cell. The flotation was performed in two stages, a roughing stage with 5 min of frother collection followed by a scavenging stage with 6 min of frother collection. Along each test, air flow of 8 L/min was injected in the roughing stage and 10 L/min in the scavenging stage. Between each flotation stage, Cytec X-133 frother was added. As a main conclusion, flotation would be feasible to re-process the tailings, allowing for the recovery of more than 70% of the arsenic present. Besides that, the flotation tails are enriched in tungsten with lower arsenic content allowing for its recovery by hydrometallurgy.

Then, alkaline extraction tests of tungsten were performed using Na_2_CO_3_ or NaOH. The last reagent proved to be more efficient. Operating conditions were optimized after performing 28 tests in a pressure reactor. The more efficient operation conditions were the following: temperature (212 °C), pressure (15 bar), chemical reagent (NaOH), residence time (2 h), liquid/solid ratio (1:1), speed rotation (400 rpm), and molar ratio NaOH/FeWO_4_ of 50.

The tests were performed in two stages with sodium hydroxide. First, the tests were carried out with pre-flotation material in order to optimize the operating conditions. In this stage, it was possible to achieve a tungsten recovery between 80 and 90% while up to 28% of the initial arsenic content remained in the leach liquors. In a second stage, post-flotation material was used along with the chemical reagent that promoted better results in the optimized operating conditions. Between 85 and 94% of the tungsten was extracted during this stage while up to 63% of the arsenic content remained in the leach liquor.

The process of zinc concentration was limited by the content of iron and arsenic in the sample material. Leaching tests were more effective when performed with sulfuric acid but the recovery was only up to 50%. The alkaline extraction yield was 94%.

The results clearly indicate that the applied methodology allows not only a higher recovery for tungsten but also a higher removal of arsenic compounds from tungsten, which is present in the tailings with very high grade.

The main issues from the reprocessing process comes from the low tonnage of W, Cu, and Zn content in the tailings; high capital costs; and foreseen high processing costs. An uncertainty remains concerning the arsenic problem and if it will be solved with reprocessing.

## Conclusions

The Cabeço do Pião tailings represent a serious environmental problem that requires a solution. It could be considered as possible the following alternatives:

1. Re-mining with reprocessing. *Advantages*: high grades in W, Cu, and Zn. *Disadvantages*: low tonnage, high capital costs, foreseen high processing costs. *Uncertainties*: Is the As problem completely solved? New sealed deposition would be needed for arsenic.
2. Cover on-site of the tailings. *Advantages*: avoids leaching and specially weathering with incorporation of As into the sediments. *Disadvantages*: topographical issues implying a complete reshape of the disposal. Large movement of ground in a difficult topography. *Uncertainties*: several solutions are possible: (a) complete cover on-site; (b) transportation of tailings and covered storage in another location.
3. Excavation of the tailings, followed by transport to another location followed by confinement. *Advantages*: sealing of the tailings becomes possible. *Disadvantages*: large volume of materials that need to be transported; reshape of the actual facility after the removal of the tailings? The reshaping is easier in the previous case. *Uncertainties*: choice of the new area in the vicinities. Necessity of an impervious bottom.
4. Excavation of the tailings followed by inertization at another location. Several solutions are possible: cementation, solidification, and polymeric resins. *Advantages*: inertization of the tailings. *Disadvantages*: large volume of materials that need to be transported; choice of the new location; and cost of the process.
5. In situ inertization. *Advantages*: avoids transport and human contact. *Disadvantages*: Is the solution feasible? Is there enough porosity? *Uncertainties*: several solutions are possible (cement, clays, polymeric resins, and geochemical immobilization).

Several alternatives have already been considered for the Cabeço do Pião tailings deposit, but not without some drawbacks. With regard to reprocessing the tailings, the low tonnage of valuable metals in the tailings and the cost of the recovery process are the main constraints. In this research, the re-mining alternative was a priority and the main subject. It is possible to conclude that in the volume of 731,034 m^3^ of fine tailings available to feed the reprocessing circuit, the average zinc content was near 1 kg t^−1^ and the mean tungsten content was 3.7 kg t^−1^. On the other hand, the average grade of arsenic was 143.9 kg t^−1^. It would possible to recover 60% of the zinc in the leaching liquor that could be further processed by electrodeposition. The proposed process would allow to produce a concentrate of arsenic with 72% of the original and reducing 43% the volume of tailings. This arsenic concentrate, with an average grade of 18.26%, would require a new sealed special storage. Finally, the pressure leaching of tungsten would allow to recover 94% of the critical metal in a liquid phase that would require further processing.

For the environmental solutions, all alternatives will have advantages and disadvantages but the ones avoiding excavation and transport will be preferable.
